# Memristor-based model of neuronal excitability and synaptic potentiation

**DOI:** 10.3389/fnins.2024.1456386

**Published:** 2024-11-18

**Authors:** Ivan M. Kipelkin, Svetlana A. Gerasimova, Alexey I. Belov, Davud V. Guseinov, Alexander V. Kruglov, Dmitry A. Serov, Max O. Talanov, Alexey N. Mikhaylov, Victor B. Kazantsev

**Affiliations:** ^1^Laboratory of Stochastic Multistable Systems, National Research Lobachevsky State University of Nizhny Novgorod, Nizhny Novgorod, Russia; ^2^Institute of Nanotechnologies Electronics and Equipment Engineering, Southern Federal University, Taganrog, Russia; ^3^Department of Engineering, University of Messina, Messina, Italy; ^4^Institute for Artificial Intelligence R&D of Serbia, Department Smart Manufacturing, Novi Sad, Serbia

**Keywords:** memristor, device, neuron, FitzHugh-Nagumo generator, ion channels, synaptic potentiation

## Abstract

In this manuscript, we investigate the memristor-based implementation of neuronal ion channels in a mathematical model and an experimental circuit for a neuronal oscillator. We used a FitzHugh-Nagumo equation system describing neuronal excitability. Non-linearities introduced by the voltage-gated ion channels were modeled using memristive devices. We implemented three basic neuronal excitability modes including the excitable mode corresponding to a single spike generation, self-oscillation stable limit cycle mode with periodic spike trains and bistability between a fixed point and a limit cycle. We also found the spike-burst activity of mathematical and experimental models under certain system parameters. Modeling synaptic transmission, we simulated postsynaptic response triggered by periodic pulse stimulation. We found that due to the charge accumulation effect in the memristive device, the electronic synapse implemented a qualitatively bio-plausible synapse with a potentiation effect with increasing amplitude of the response triggered by a spike sequence.

## 1 Introduction

Neuromorphic systems reproducing neuronal circuits and functions of the brain have attracted growing attention of researchers from different fields of science and technology. Spiking neuronal networks employ memristive devices to implement neuronal and synaptic components. Engineering of spiking neuronal networks and corresponding processing functions nowadays look as one of the most intriguing directions in neuromorphic system development (Makarov et al., 2022; Dalgaty et al., 2024).

Memristors are electronic components based on the resistive switching (RS) effect (Chua, 2019), possessing at least two stable states that differ in their own resistance—a state with high (HRS) and low (LRS) electrical resistance. Devices utilizing this effect retain the acquired information in the form of resistance, the evolution of which is characterized by the restructuring of the atomic structure in thin insulating (dielectric) layers of nanometer-scale thickness under the stimulus of an electric field. Employing memristors as elements in electronic circuits have opened wide possibilities of designing non-linear oscillators with a variety of complex dynamical modes including chaos and multistability (Minati et al., 2020; Gokyildirim et al., 2022; Wang et al., 2022; Boudjerida et al., 2023; Chen et al., 2019; Spagnolo et al., 2022; Corinto and Forti, 2017). Possibility of complex non-linear dynamics and unique (biomimmetic) electro-physical properties with energetic efficiency has made memristors to be the most promising candidates for constructing biologically plausible neuron models and neuromorphic computations (John et al., 2022; Indiveri et al., 2013; Pisarev et al., 2020; Shchanikov et al., 2021). Specifically, memristors were used to simulate the dynamics of voltage-gated ion channels of neuron membrane. Implementation of rather simple potassium channels was discussed in Najem et al. (2018), Thomas (2013), Yi et al. (2018), and Gonzalez-Raya et al. (2020). More complex Hodgkin-Huxley neuron model employing both sodium and potassium channels was realized in Lv et al. (2016), Jeong et al. (2016), Sah et al. (2014), Gonzalez-Raya et al. (2019), and Hu and Liu (2019). Our recent study was reported on memristor-based implementation of FitzHugh—Nagumo (FHN) spiking neuron model that can reproduce both excitable and oscillatory neuronal dynamics (Kipelkin et al., 2023).

Synaptic plasticity is one of the fundamental properties of living neuronal systems responsible for basic cognitive functions of the brain such as learning and memory (Sun et al., 2024; Kotaleski and Blackwell, 2010). Signals are transmitted between neurons via special biological devices called synapses. The strength of the synaptic connection is defined by complex chemical molecular transformations that occurs in both presynaptic (transmitter) neuron and postsynaptic (receiver) neuron (Lynch, 2004). Specifically, long-term changes in the connection strength are localized mostly in the postsynaptic neuron. When a spike is transmitted, the postsynaptic membrane, similarly to basic neuronal excitability, opens its ion channels and ions, particularly *Na*^+^ and *K*^+^, cross the membrane generating postsynaptic potentials (Nadler, 2012). Interestingly, that is, the synapse is transmitting a series of; each consequent spike may induce voltage responses of variable amplitudes. If each next spike generates a stronger response of increasing amplitude, then the *synaptic* *potentiation* takes place (Vyazovskiy et al., 2008). So, the synaptic connection amplifies its strength. In the opposite case, there is a synaptic depression. The type of synaptic plasticity is defined by the neuron type and also by the dynamical characteristics of the transmitted signals, for example, frequency of spike and/or relative phase of the spike occurrences.

For neuron models describing ion channels, information processing and encoding can be described by the dynamics of the action potential. However, due to the stochasticity and sensitivity of memristors, which affect dynamic processes, more in-depth mathematical and experimental studies are needed to control the behavior of devices and prevent undesirable effects through operational control.

Based on the above sources and existing problems, we investigated a 3D model of neuronal excitability, implemented by two memristor-based FHN generator circuit. We employed memristive devices with different electrode compositions *Au*/*Ta*/*ZrO*_2_(*Y*_2_*O*_3_)/*Pt*/*Ti*/*glass* and *Au*/*Ru*/*ZrO*_2_(*Y*_2_*O*_3_)/*Pt*/*Ti*/*glass* to mimic ion channels. By extending the equations and modifying the scheme proposed earlier (Kipelkin et al., 2023), we presented mathematical and experimental investigation of the model. Experimental data obtained as a result of hardware measurements qualitatively confirm the computational modeling. We also analyzed how the electronic neuron responded on a spike sequence. Similarly to a postsynaptic neuron in real neuronal networks, our memristor-based device demonstrated synaptic potentiation when each next spike induced the response of growing amplitude.

## 2 Materials and methods

### 2.1 Memristive devices

**Figure 1:**
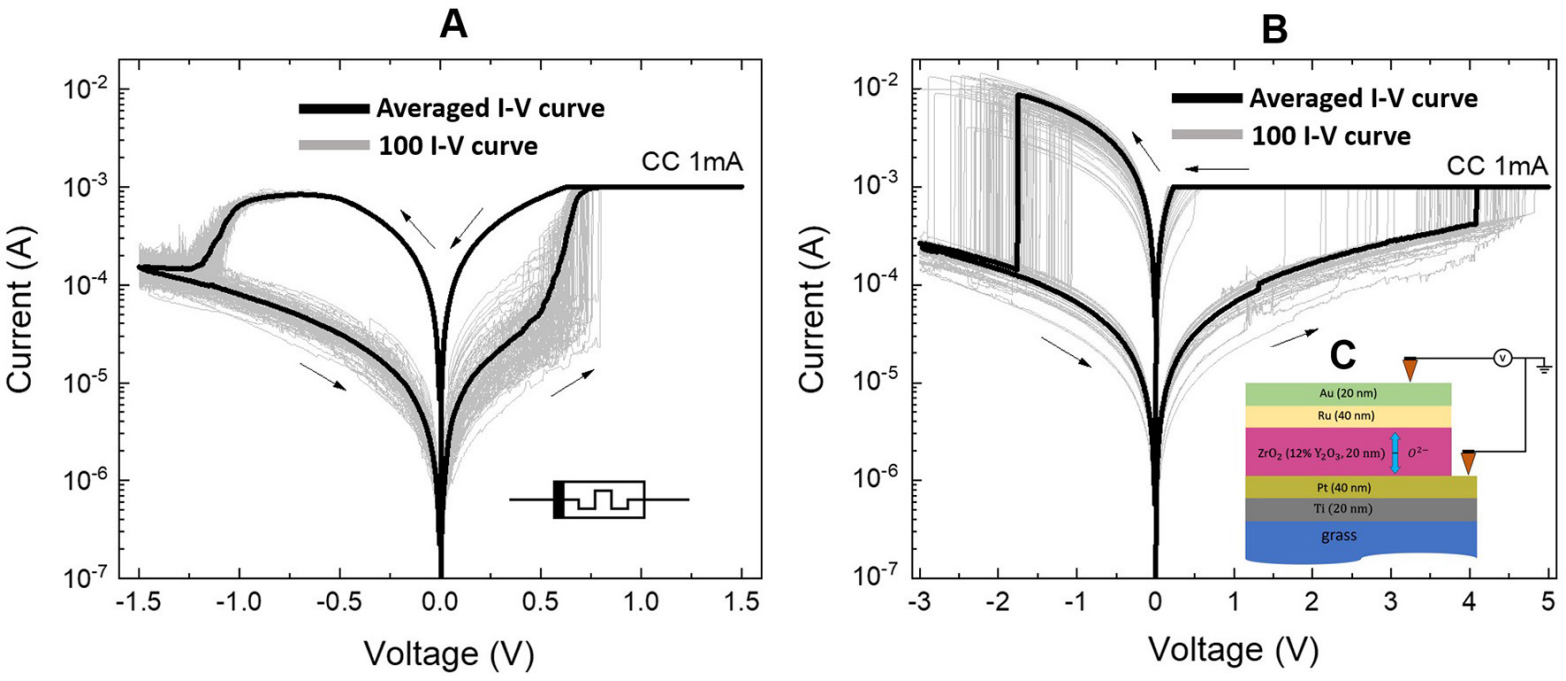
Experimental current-voltage characteristics of non-volatile devices. **(A)** *Au*/*Ta*/*ZrO*_2_(*Y*_2_*O*_3_)/*Pt*/*Ti*/*glass*, **(B)** *Au*/*Ru*/*ZrO*_2_(*Y*_2_*O*_3_)/*Pt*/*Ti*/*glass*. Arrows show the voltage sweep. The number of characteristics taken was 100 pcs. **(C)** Schematic cross section of the *Au*(20*nm*)/*Ta*(40*nm*) or *Ru*(40*nm*)/*ZrO*2(*Y*)(20*nm*)/*Pt*(40*nm*)/*Ti*(20*nm*)/*grass* memristive device.

To simulate neuron ion channels, we used two non-volatile memristive devices of the bipolar switching type having different electrodes. Figure 1 illustrates the experimental current-voltage characteristics on a logarithmic scale along the ordinate axis of the multistable devices *Au*/*Ta*/*ZrO*_2_(*Y*_2_*O*_3_)/*Pt*/*Ti*/*glass* and *Au*/*Ru*/*ZrO*_2_(*Y*_2_*O*_3_)/*Pt*/*Ti*/*glass*, respectively, with different metal contact interfaces that demonstrate reproducible bipolar switches between the LRS and HRS states.

To create memristive devices on a glass substrate, Torr International's 2G1-1G2-EB4-TH1 vacuum film deposition system and standard photolithography equipment were used. *ZrO*_2_ films were deposited using high-frequency magnetron sputtering from a target made from a mixture of *ZrO*_2_ (0.88 shares) and *Y*_2_*O*_3_ (0.12 shares) powders at a temperature of 250 °C. The metal electrode layers were then applied using magnetron sputter at direct current and 200 °C. The *ZrO*_2_ film has a thickness of 20 nm. The top electrode was a 20 nm *Au* film with a 40 nm *Ta* or *Ru* sublayer. The bottom electrode was *Pt* film with a thickness of 40 nm and *Ti* sublayer, that is, 20 nm thick. Detailed information on the technological processes can be found at Khan et al. (2019), Gorshkov et al. (2016), Yildirim and Pachter (2019), Mikhaylov et al. (2015), Mikhaylov et al. (2020), and Baranova et al. (2020).

Identification of electrical parameters in continuous and pulse modes was carried out using an Agilent B1500A semiconductor analyzer with a sweeping rate of 7.25 V/s. Connections to contact pads of the device were carried out using the Everbeing EB-6 probe station. We used current compliance (CC) of 1 mA. We took all measurements at room temperature and normal atmospheric pressure. The voltage offset on the device corresponds to the potential difference between the top electrode (*Au*) and the grounded bottom electrode (*Pt*).

We used *Ru* and *Ta* top electrodes with differences in resistive switching parameters in our memristive devices. In particular, the *R*_*HRS*_ of the memristive devices on the *Ru* and *Ta* electrode corresponds to ≈10 − 12 *k*Ω. The *R*_*LRS*_ values ≈700 Ω for *Ta* and ≈200 Ω for *Ru*. The process of RS is closely linked to the diverse materials used for the top electrode, the mechanisms of switching, and the nature of electronic transport. Various metals exhibit different oxygen affinities, leading to distinct redox reaction processes and the formation of defects in metal oxides. We can estimate the approximate values of switching dynamic ranges using the values above for the electrode using *Ta*: $\frac{R_{HRS}}{R_{LRS}}$ = 20 and for the electrode using *Ru*: $\frac{R_{HRS}}{R_{LRS}}$ = 60. Our experiments indicate that memristive devices have different resistance values in different resistive states. This range of values can vary from cycle to cycle as well as from device to device. The variation in the characteristics of memristive devices was taken into account as follows. In our experiments, we observed a random value of the switching voltage (*V*_*set*_) during initial measurements. This observed value was applied to the device in subsequent measurements. Each time a switching cycle occurred, we introduced a random perturbation to the *V*_*set*_ threshold. The distribution of this random variable can be modeled using a normal distribution. This approach is also applicable to other relevant parameters, including the switching voltages, memristor resistances in the HRS and LRS, as well as the potential barriers for ion and electron transport. At the same time, the devices had reproducible characteristics for neuromorphic computing, possessing stable and gradual resistive switching of the bipolar type (Gorshkov et al., 2014; Hu et al., 2024). The corresponding voltage changes as SET and RESET transitions were given with interval values for the electrode based on *Ru*: *V*_*set*_ = 4 V, *V*_*reset*_ = −2 V, and for electrode based on *Ta*: *V*_*set*_ = 1 V, *V*_*reset*_ = −1.4 V. Statistical data on the memristive devices under study are presented in Supplementary material.

### 2.2 Mathematical model

The dynamics of neurons was determined by a modified FitzHugh-Nagumo (mFHN) model. This model is based on the assumption that ion currents associated with nerve pulse conduction can be divided into fast-acting and slow-acting components. These components are responsible for generation, adhesion, and rest, respectively, of the membrane. The mathematical description of the model is introduced by the equations obtained and investigated in Binczak et al. (2006):


(1) $$\begin{array}{l} \left\{ \begin{array}{l} \overset{\cdot }{u}=F_{1,2}\left(u\right)-\vartheta +W_{ex}\\ \overset{\cdot }{\vartheta }=\varepsilon \cdot \left[g\left(u\right)-\vartheta -\eta \right] \end{array}\right., \end{array}$$

The fast variable *u* describes the membrane voltage of the neuron. ϑ qualitatively describes the dynamics of slow (potassium) currents. *W*_*ex*_ is an external pulse current with a certain duty cycle (*Q*) and amplitude (*A*_*ex*_), respectively. Function *g*(*u*) is a piecewise linear function determined by the polarity of the voltage on the neuron membrane, *g*(*u*) = α·*u* for *u* < 0 and *g*(*u*) = β·*u* if *u*≥ 0, where α=0.78 and β=1.86 are the constants that determine the dynamics of the recovery ϑ. ε is the parameter that controls the level of depolarization and therefore determines the dynamic model of the neuron, and η is a constant parameter. We have added a detailed description of the derivation of Equation 1 and placed it in the Supplementary material.


(2) $$\begin{array}{l} F_{1,2}\left(u\right)=\gamma_{1}\cdot I_{1}\cdot d_{1}+\gamma_{2}\cdot I_{2}\cdot d_{2} \end{array}$$

The function presented in Equation 2 is a non-linear function defined as the sum of the product of the current in the memristor and its active load *d* (load resistance). Coefficient γ_1, 2_ is determined from the method of the least squares approximation of current-voltage characteristic and having the dimension *V*^−1^. The indices 1 and 2 denote *Au*/*Ta*/*ZrO*_2_(*Y*_2_*O*_3_)/*Pt*/*Ti*/*glass* and *Au*/*Ru*/*ZrO*_2_(*Y*_2_*O*_3_)/*Pt*/*Ti*/*glass* devices, respectively. The current itself consists of the product of the current density and the fixed area of the electrodes (*S*_*el*_) of the memristor, *I*_1_ = *j*_1_·*S*_*el*_.

Mathematical expressions for the current densities were used in the following form (Chua and Kang, 1976) (Equations 3–5).


(3) $$\begin{array}{l} j_{1,2}=x_{1,2}\cdot j_{lin_{1,2}}+\left(1-x_{1,2}\right)\cdot j_{nonlin_{1,2}} \end{array}$$

(4) $$\begin{array}{l} \left\{ \begin{array}{l} j_{lin_{1,2}}=|u|\cdot \sigma_{1,2}^{-1}\\ j_{nonlin_{1,2}}=|u|\cdot B\cdot exp\left(b_{1,2}\cdot \sqrt{\left|u\right|}-E_{b_{1,2}}\right) \end{array}\right. \end{array}$$

(5) $${\overset{\cdot }{x}}_{1,2}=\{\begin{array}{l} A\;\cdot \;exp(-E_{m_{1,2}}-\delta_{1,2}\;\cdot \;u)\cdot f_{1,2}(x_{1,2},p),u>V_{set}\\ 0,V_{reset}<u<V_{set}\\ -A\;\cdot \;exp(-E_{m_{1,2}}+\delta_{1,2}\;\cdot \;u)\cdot f_{1,2}(x_{1,2},p),u<V_{reset} \end{array}$$

where *j*_1, 2_ is the current density going through the memristor, which consists of a linear component (*j*_*li*_*n*__1, 2__) and a non-linear component (*j*_*nonli*_*n*__1, 2__), σ_1, 2_ is a specific resistance, *u* is external voltage acting on the input of device, and *B, A, b*_1, 2_, δ_1, 2_ are constants determined from experimental data (from the approximation of the current-voltage characteristics of the corresponding device). Energy values *E*_*b*_1, 2__, *E*_*m*_1, 2__ denote effective barriers for electron jumps and oxygen ion hopping, respectively. State variable *x*_1, 2_ has a probabilistic nature describing how current density can randomly change in the range from 0 to 1. When *x*_1, 2_ = 1, the memristive device is highly conducting (low resistance state). When *x*_1, 2_ = 0, the memristive device is in the high resistance state, and the change in this quantity (*x*_1, 2_) corresponds to the drift velocity of ions in the dielectric layer, exponentially dependent on the applied voltage to the device.

To correctly implement (Equation 5), we introduced the window function using (Joglekar and Wolf, 2009).


(6) $$\begin{array}{l} f_{1,2}\left(x_{1,2},p\right)=1-{\left(2\cdot x_{1,2}-1\right)}^{2p} \end{array}$$

The proposed window function restricts the dynamic (Equation 6) in an acceptable range, satisfying the boundary conditions, *f*(0) = *f*(1) = 0 for all positive values of *p*. Equations describing the variable *x*_1, 2_ derive from the diverse internal processes of filament development in our memristive device. A detailed description of parameters is presented in Mishchenko et al. (2022), and the values are in Supplementary material.

Summarizing Equations 1–6 we obtained the following 3D system of differential equations:


(7) $$\begin{array}{c} \overset{\cdot }{u}=|u|\underset{1,2}{\sum }\gamma_{1,2}\cdot [x_{1,2}\cdot \sigma_{1,2}^{-1}+\left(1-x_{1,2}\right)\cdot B\cdot exp(b_{1,2}\\ \cdot \sqrt{\left|u\right|}-E_{b_{1,2}})]\cdot S_{el_{1,2}}\cdot d_{1,2}-\vartheta +W_{ex}\\ \overset{\cdot }{\vartheta }=\varepsilon \cdot \left[g\left(u\right)-\vartheta -\eta \right] \end{array}$$

$${\overset{\cdot }{x}}_{1,2}=\{\begin{array}{l} A\;\cdot \;exp(-E_{m_{1,2}}-\delta_{1,2}\;\cdot \;u)\cdot f_{1,2}(x_{1,2},p),u>V_{set}\\ 0,V_{reset}<u<V_{set}\\ -A\;\cdot \;exp(-E_{m_{1,2}}+\delta_{1,2}\;\cdot \;u)\cdot f_{1,2}(x_{1,2},p),u<V_{reset} \end{array}$$

Integration of Equation 7 was implemented using a built-in MATLAB solver of ordinary differential equations based on the Runge—Kutt (RK4) algorithm with the following fixed parameters: integration error: ψ = 10^−10^; constant step: *s* = 0.02 and initial conditions (-0.65, 0, 0.00001, 0.00001).

### 2.3 Experimental model

**Figure 2:**
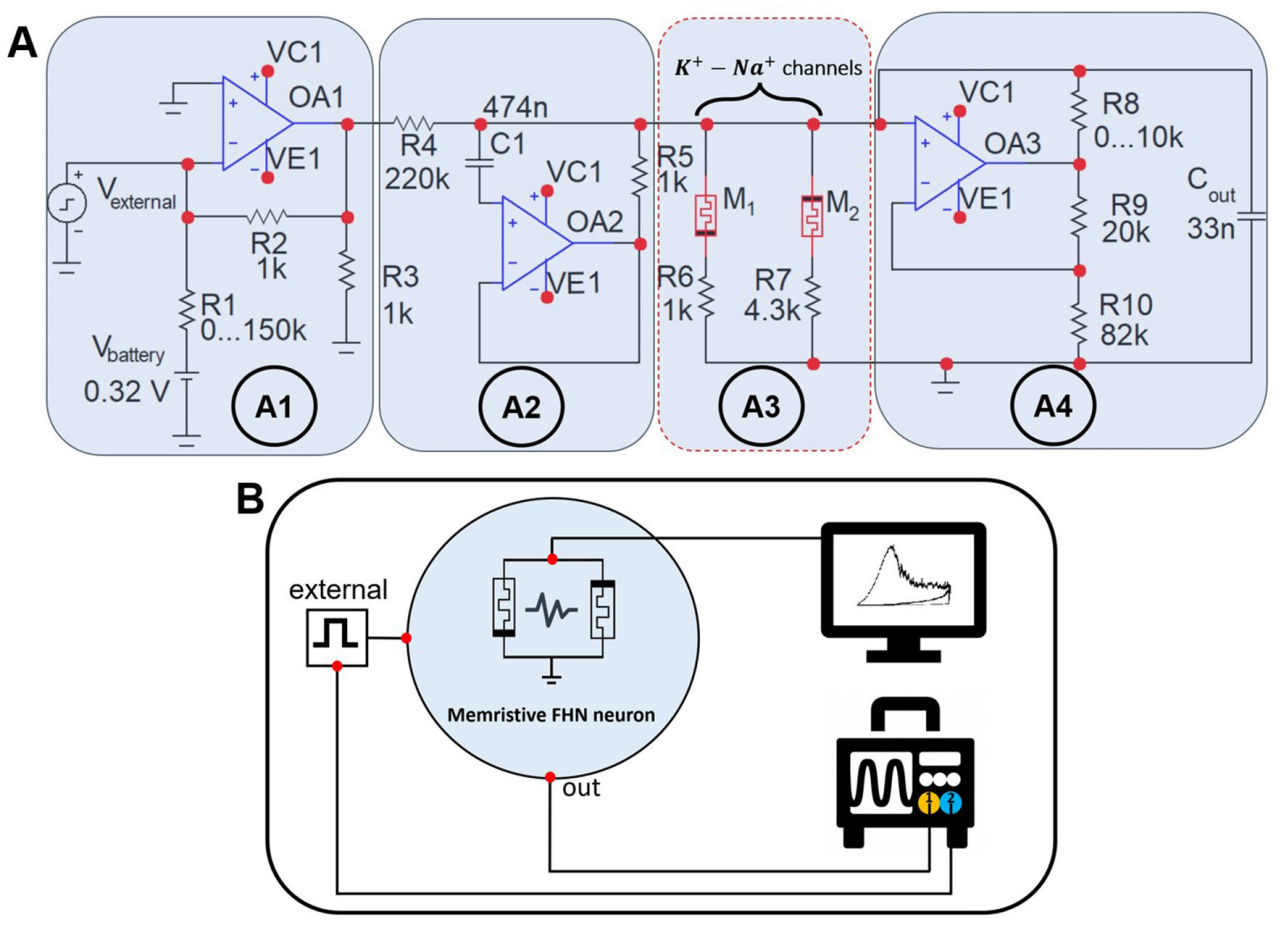
System for conducting an experiment on non-volatile memristive devices. **(A)** The experimental mFHN model circuit is based on memristive devices that simulate sodium and potassium ion channels in biological neurons. Memristor *M*_1_ is based on the *Au*/*Ta*/*ZrO*_2_(*Y*_2_*O*_3_)/*Pt*/*Ti*/*glass* device, and *M*_2_ describes the *Au*/*Ru*/*ZrO*_2_(*Y*_2_*O*_3_)/*Pt*/*Ti*/*glass* memristive device. *R*_6_ and *R*_7_ are load resistances for the corresponding memristive devices. **(B)** Schematic illustration of experimental setup recording signals from the memristive neuron electronic circuit. Processing and visualization of the recorded transient processes is carried out using the OriginPro 2019b software.

Figure 2A illustrates experimental circuit simulating a postsynaptic neuron modeled by mFHN (Gerasimova et al., 2021; Kipelkin et al., 2023; Binczak et al., 2006). The circuit includes a counter-parallel connection of two memristors *M*_1_ and *M*_2_ simulating *Na*^+^ and *K*^+^ ion channels of neuronal membrane, respectively (Figure 2A3). The input block (Figure 2A1) of the circuit consists of a power source, which is a 1.5 Volt battery (*V*_*battery*_), and a potentiometer *R*_1_ ∈ [0;150] *kΩ*, which is used to switch dynamic modes. The input block also includes a source of external stimulus (*V*_*external*_), created by the KEYSIGHT 33600A random pulse generator, which has a 14-bit capacity and sampling frequency of 120 MHz. Next, the signal propagates through the circuit and enters the coupling *RL* generator (Figure 2A2) implemented with the MC1458L operational amplifier with a resistance value of *R* = *R*_5_ and inductance *L* = *C*_1_*R*_4_*R*_5_. The output block (Figure 2A4) includes a capacitor *C*_*out*_, which creates a voltage drop across the channels, and an adjustable variable resistor *R*_8_ ∈ [0;10] *kΩ* which changes the amplitude and duration of oscillations at the output. The entire process of signal flow can be considered as the accumulation of potential on the neuron membrane.

The block diagram of the experimental setup is shown in Figure 2B. Agilent B1500a analyzer is used to analyze the characteristics of memristive devices. The signal generated by the analog neuronal oscillator was recorded on the *C*_*out*_ capacitor and then read using 1 channel by an ALFATEC S7-334 digital three-channel oscilloscope with a sampling rate of 5 GHz. The inbound signal from the external stimulus was displayed in channel 2.

## 3 Results

### 3.1 Simulations of the mathematical model

**Figure 3:**
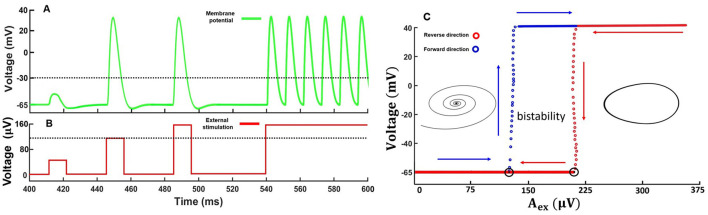
Results of computational modeling on the response of a memristive neuron to external impulse stimulation. **(A)** Membrane potential response to a single subthreshold, threshold, and suprathreshold signals. The threshold value of the membrane potential is −30*mV*. **(B)** Type of stimulation of the membrane potential. The dotted line indicates the pulse generation threshold. Pulse duration in the first three cases was 11 ms. Model parameters: ε = 0.009, η = 0.1. **(C)** Bifurcation diagram illustrating the excitable, bistable, and self-oscillatory modes of the memristive neuron, filmed during forward and reverse passage of external stimulus. The diagram on the left shows an example of a trajectory on the plane (*u*, ϑ) for the excited mode and on the right for the self-oscillating mode.

First, we consider how the memristive neuron modeled by Equation 7 responded to stimulation pulses of different amplitudes (*A*_*ex*_). Figures 3A, B illustrate the results of computational modeling. Increasing of the pulse amplitude upper certain threshold led to generation of the response pulse. The pulse shape was qualitatively similar to neuronal excitability mediated by sodium and potassium transmembrane currents.

With prolonged external stimulus, the neuron switched to a self-oscillatory mode and generated a periodic sequence of pulses, qualitatively similar to regular oscillations of the membrane potential in real neurons of the 3rd excitability class (Izhikevich, 2007). Note that the model dynamics demonstrates the qualitative match of the main characteristics and properties of the pulses with biological action potential properties, including the existence of a threshold leading to an "all-or-none" response, as well as the presence of refractory periods.

In addition, the amplitude of the external impact determines one of the three possible dynamic modes of the memristive neuron including excitable, bistable, or oscillatory ones. According to the bifurcation diagram in Figure 3C, a region of bistability emerges in the dynamics of the memristive neuron (Equation 7). Bistability, in this case, means that, depending on the initial conditions of the system, for example, ($u^{0},\vartheta^{0},x_{1}^{0},x_{2}^{0}$), the neuron can either maintain a resting potential or generate a periodic sequence of spikes. In other words, in the phase space of the dynamical system given by Equation 7, a stable fixed point coexists with a stable limit cycle. We found that for the set of parameters used, the bistability region appeared in the range of voltages 120 μV ≤ *A*_*ex*_ ≤ 200 μV. The excitable mode, in which the resting potential is stable, and when the threshold is exceeded, one or more pulses are generated is realized at *A*_*ex*_ < 120 μV. For *A*_*ex*_> 200 μV, the resting potential loses stability and self-oscillations emerge in the system via a subcritical bifurcation scenario. Corresponding stable limit cycle attracts all trajectories representing only one attractor. Bifurcation point *A*_*ex*_=120 μV corresponds to the fold limit cycle bifurcation. In the case of increasing voltage, the 2-fold limit cycle is divided into robust stable and unstable limit cycles. The unstable limit cycle disappears at *A*_*ex*_=200 μV via subcritical Andronov-Hopf bifurcation. According to Izhikevich's classification, our neural model can be defined as a bistable resonator (Moehlis, 2008).

**Figure 4:**
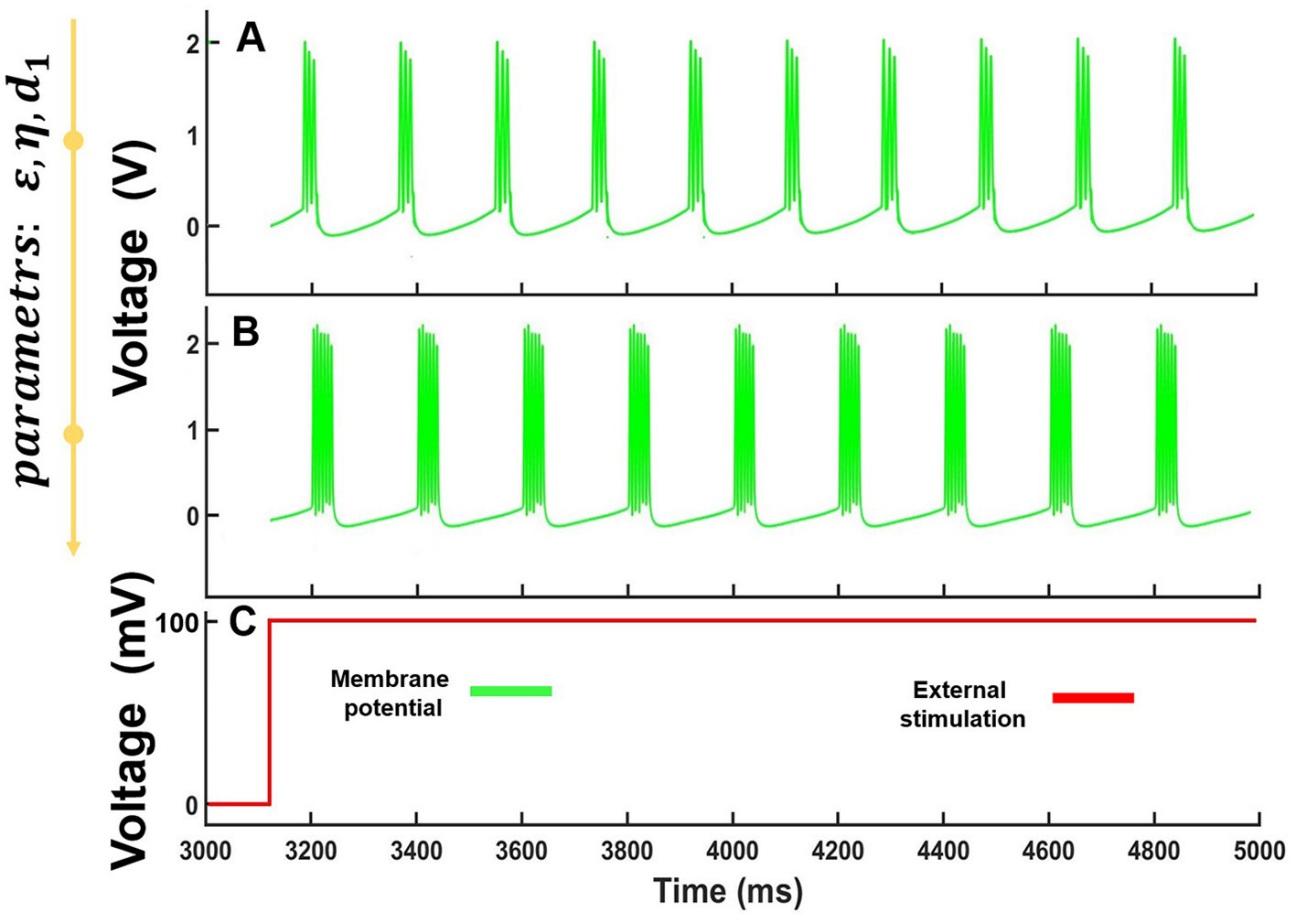
Computational modeling of Equation 7 for a constant voltage level stimulation. **(A)** Bursts composed of three spikes for ε= 0.0028 , η = 0.068 and *d*_1_= 875, **(B)** Bursts composed of six spikes for ε= 0.0049 , η = 0.074 and *d*_1_ = 927. The fixed value is *d*_2_= 4,300. **(C)** The form of stimulating the form of stimulating voltage.

Interestingly, the specific parameter range of the model demonstrated spike-burst oscillations. In the case of ε∈ [0.001;0.006], η∈ [0.06;0.08], *d*_1_∈ [840;985] and for fixed *d*_2_ = 4300, we observed the spike-burst dynamics with a different number of spikes in the burst as illustrated in Figure 4.

### 3.2 Electronic circuit experimental study

**Figure 5:**
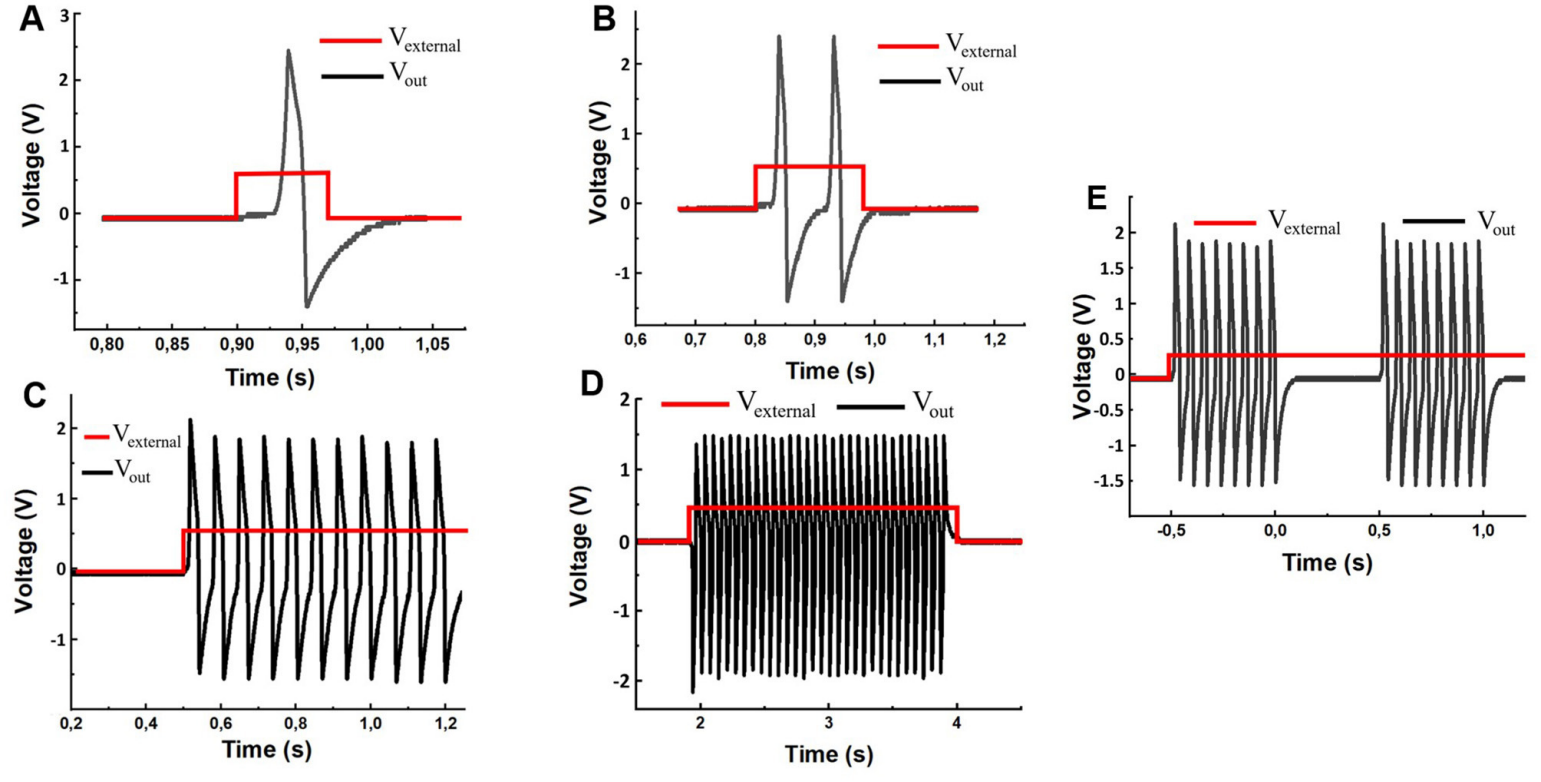
Electronic circuit waveforms driven by stimulation square pulses with a given duration and a fixed amplitude of 120 millivolts. **(A)** Single pulse response to a short stimulation of 0.074-s duration. **(B)** Two consequent spikes in response to the 0.18-s stimulus. **(C)** Multiple spike responses to the 2-s stimulus. **(D)** The constant level depolarization yields a periodic spike sequence. **(E)** The experimentally spontaneous spike-burst oscillations for *R*_1_ = 121 kΩ, *R*_8_ = 7.8 kΩ and 120 mV external stimulation voltage.

After checking the performance of the generator (using the system in Figure 2), by calibration, we applied an external unipolar time-dependent effect to the input and changed the values of the *R*_1_ potentiometers in the range from 0 to 100 *kΩ* and *R*_8_ from 0 to 5 *kΩ*. As a result, we observed a variety of output signals similar to typical biological neuron responses including both single and multiple activities with a certain number of spikes, as shown in Figure 5.

**Figure 6:**
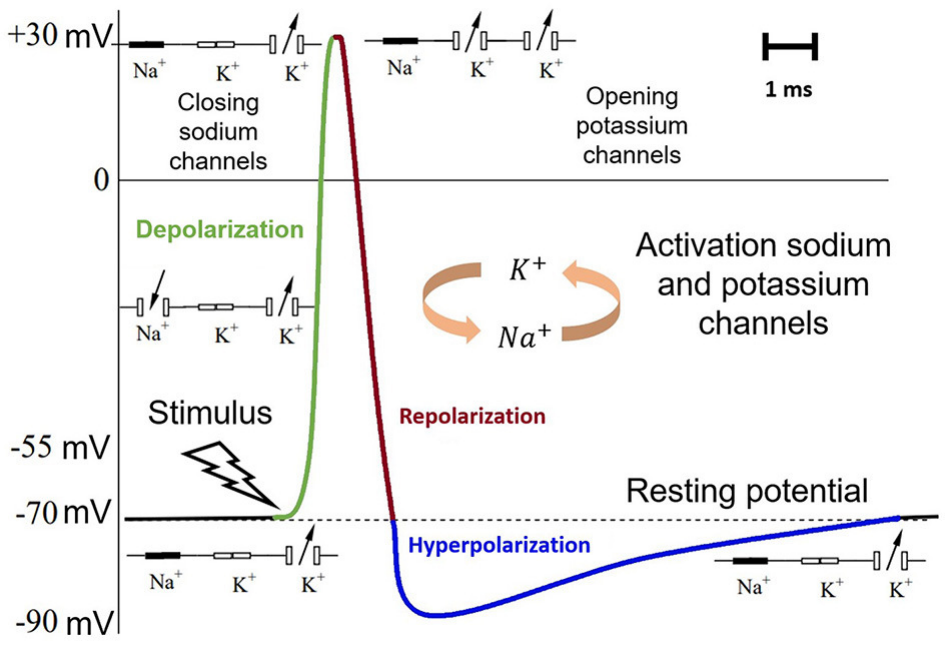
Mechanism of spike generation. The resting potential is indicated in black. The depolarization—green. The repolarization—dark red. The hyperpolarization in blue.

The mechanisms of spike generation in the memristor-based circuit (Figure 2A) can be qualitatively described similarly to the processes of depolarization, repolarization, and hyperpolarization as follows (Figure 6).

The conductive filaments inside the memristive devices were induced by the same voltage *U*. The counter-parallel connection of the devices ensures the flow of current in the opposite direction. If the voltage *U* was low enough (under 100 millivolts), then the current passed through only one memristor (*M*_1_). Consequently, the capacitor (*C*_*out*_) receiving this current was slowly charging (*hyperpolarization*). Gradually increasing the voltage *U* to a certain threshold value (~120 millivolts), the second memristor (*M*_2_) also began to conduct current that went completely to the capacitor causing a voltage jump (*depolarization*). In turn, the first memristor started to discharge the capacitor receiving a strongly negative current reducing its voltage *U* to zero (*repolarization*), after which the process can start again. This whole process was qualitatively analogous to the voltage avalanches in biological neurons caused by the successive opening and closing of the ion channels *Na*^+^ and *K*^+^.

With a further increase in the values of potentiometers *R*_1_ and *R*_8_, well as the replacement of the load resistance *R*_6_ = 700 Ω (with fixed *R*_7_ = 4.3 kΩ), the memristive neuron generated spontaneous spike-burst oscillations (Figure 5E) in response to a constant pulse of external stimulation. The first spike was caused by external stimulation, while subsequent spikes were formed spontaneously due to the intrinsic stochastic nature of the memristive devices and continue with constant voltage drive. Note that the combination of two memristive devices working in counter-parallel was crucial to generate the spike-burst oscillations. If only one memristor was used, such dynamics was not possible.

### 3.3 Simulation of synaptic potentiation

**Figure 7:**
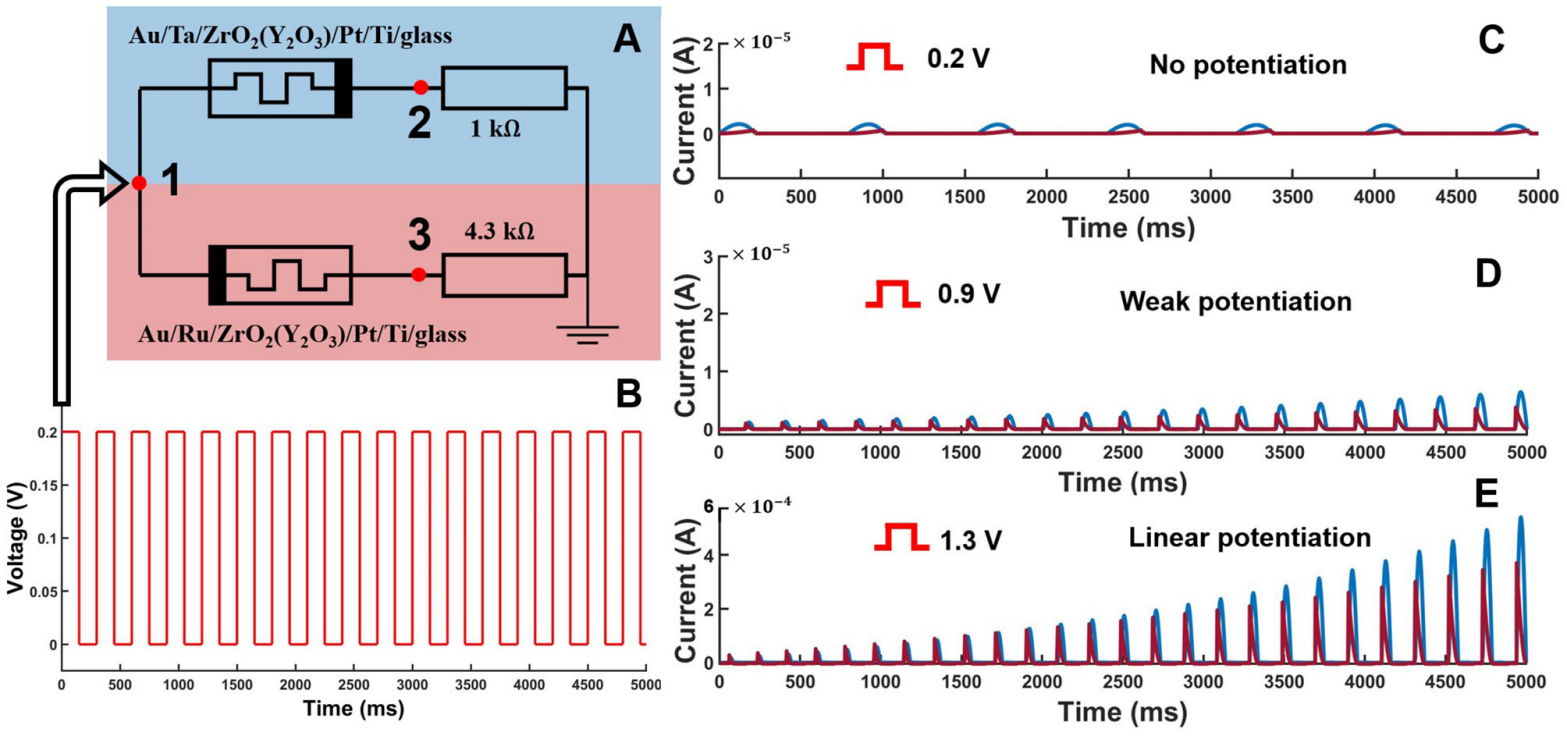
Illustration of synaptic potentiation effect in the memristive devices. **(A)** Scheme of the experiment with counter-parallel memristive devices *Au*/*Ta*/*ZrO*_2_(*Y*_2_*O*_3_)/*Pt*/*Ti*/*glass* and *Au*/*Ru*/*ZrO*_2_(*Y*_2_*O*_3_)/*Pt*/*Ti*/*glass*. A section of the whole circuit was taken from Figure 2A3. **(B)** Stimulation signal. Transient current characteristics with **(C)** absence, **(D)** weak, and **(E)** strong synaptic potentiation of memristive devices at different voltage amplitudes (shown in the figure). The blue color corresponds to the device *Au*/*Ta*/*ZrO*_2_(*Y*_2_*O*_3_)/*Pt*/*Ti*/*glass*, and the red color corresponds to *Au*/*Ru*/*ZrO*_2_(*Y*_2_*O*_3_)/*Pt*/*Ti*/*glass*.

In our neuron, two memristive devices simulating sodium and potassium ion channels determined non-linearity and excitability. Next, we checked whether such a circuit can generate a variable response, for example, the synaptic plasticity, on a series of pulse stimulations. Figure 7 illustrates simulation results of the memristive device responses on a series of pulse stimulations. A pulsatile signal was applied on the two counter-parallel memristors simulating *Na*^+^ and *K*^+^ ion channels. For relatively low amplitude of the stimulation pulses, the consequent response pulse had equal amplitudes. However, for increasing amplitude, we found that each forthcoming pulse had a larger amplitude than the previous one. In other words, the effect qualitatively similar to synaptic potentiation in neurons takes place in the memristive neuron. The memristive devices simulate the functionality of the postsynaptic part of the synaptic transmission. The larger the stimulation pulse amplitude, the higher strength of the potentiation was observed.

The stochastic nature of non-volatile memristive devices determines the mechanism of synaptic potentiation, which leads to an increase in the coupling between the memristive devices. For a qualitative analysis of the phenomenon, we considered a region of non-linearity in the circuit (Figure 7 input point 1) where a positive square pulse of various amplitudes was supplied, ranging from 0.2 to 1.3 V and with a duty cycle of 0.3. The input voltage of the memristive devices led to the appearance of the output current, taking into account the internal resistance of the devices, the values of which were taken from points 2 and 3 for the devices *Au*/*Ta*/*ZrO*_2_(*Y*_2_*O*_3_)/*Pt*/*Ti*/*glass* and *Au*/*Ru*/*ZrO*_2_(*Y*_2_*O*_3_)/*Pt*/*Ti*/*glass*, respectively. When a subthreshold voltage of ~0.2 V was applied, there was no synaptic potentiation (Figure 7C). The transient current characteristics for all periodic voltage pulses showed relatively weak activity and subsequent attenuation in time because the memristive devices remained in their original state of static conductivity. However, we can notice that as the voltage increases, there is a gradual increase in conductivity within the memristive devices that leads to minor potentiation and an increase in current characteristics over time (Figure 7D). With a sufficient level of input voltage in memristive devices, there was a steady trend toward a linear increase in the temporal characteristics of the current (Figure 7E), associated with an increase during the synaptic potentiation. We also conducted a more in-depth analysis of the postsynaptic nature of memristive devices, establishing an interesting pattern and presenting it in Supplementary material.

**Figure 8:**
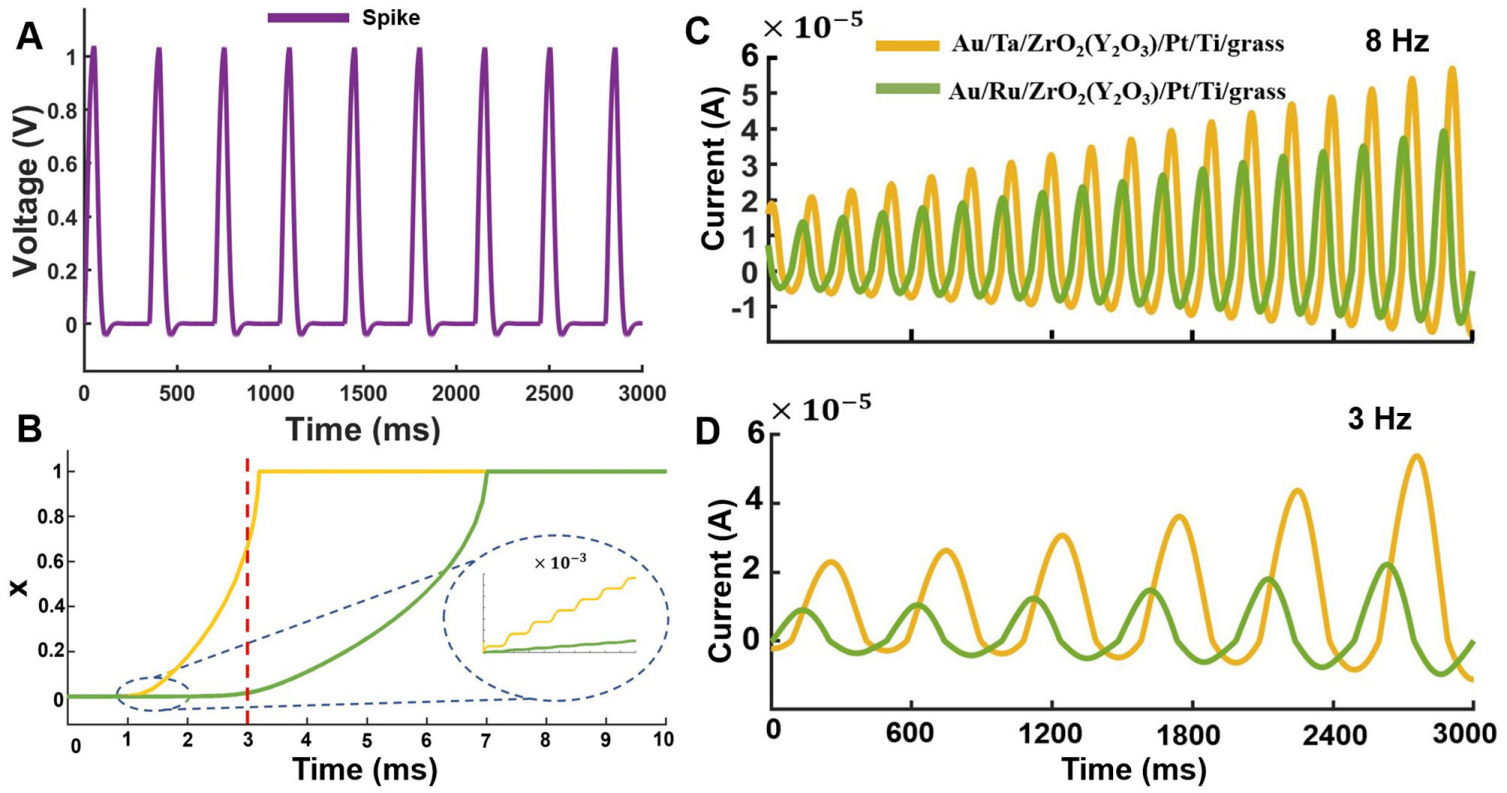
Synaptic potentiation in the memristive neuron model (Equation 7) based on two memristive devices. **(A)** An example of low-frequency spike activity with an amplitude of 1 V and a frequency of 3 Hz. **(B)** The state variable of the memristive devices (Equation 5). The inset shows an enlarged scale. The jump corresponds to the switching of the memristive device in response to a spike. Comparative characteristics of the potentiation mechanism at a frequency of **(C)** 3 Hz and **(D)** 8 Hz. The yellow color corresponds to the device *Au*/*Ta*/*ZrO*_2_(*Y*_2_*O*_3_)/*Pt*/*Ti*/*glass*, and the green color corresponds to *Au*/*Ru*/*ZrO*_2_(*Y*_2_*O*_3_)/*Pt*/*Ti*/*glass*. Detailed information about the figures is in Supplementary material.

Next, we investigated whether the entire electronic circuit (Figure 2A) based on *Au*/*Ta*/*ZrO*_2_(*Y*_2_*O*_3_)/*Pt*/*Ti*/*glass* and *Au*/*Ru*/*ZrO*_2_(*Y*_2_*O*_3_)/*Pt*/*Ti*/*glass* demonstrates the synaptic potentiation. Simulating the corresponding dynamical system (Equation 7), we found that the effect of synaptic potentiation can be induced by a sequence of low-frequency spike train as illustrated in Figure 8.

To illustrate the mechanism of synaptic potentiation, we used a split time scale derived from device state functions. According to Figure 8B, we chose a time interval of up to 3 s. In this range, the devices exhibited mixed dynamics due to the intrinsic stochastic nature and manifested through a step-exponential transition from HRS to LRS. The results of the computational modeling indicated a change in the shape of the transient characteristics of the current depends on the type of stimulation. If we used inbound spikes (Figure 8A) with negative values, the output current has also of negative values (Figures 8C, D). Note that saturation of synaptic potentiation was achieved quicker with a decrease in the frequency of stimulation, but with a less pronounced change in the conductivity of the devices (Figures 8C, D).

## 4 Discussion

To highlight the potential of our study, we present a comparative table that evaluates our proposed model against (Huang et al., 2021) (The model 1) and (Nabil et al., 2022) (The model 2), both qualitatively and quantitatively. We compared the following model parameters (Table 1):

- the structural composition of the insulator used in the memristive devices;
- mathematical model of a neuron describing the ionic dynamics of a system;
- the number of adjustable parameters in the modeling process;
- consideration is given to the internal dynamics of the memristive devices, including ion and electron transport processes within the filament;
- the mathematical model is experimentally validated using electrical circuits and physical memristive devices;
- availability of statistical data on the current-voltage characteristics of physical memristive devices with different compliance current values;
- the number of memristive devices used in the model or circuit;
- the operating voltage range of the memristive device;

**Table 1:** Qualitative and quantitative comparison of the existed neuronal ionic dynamics models.

**N**	**Items**	**Our model**	**The model 1**	**The model 2**
**1**	Insulator structure	*ZrO*_2_	*NbO*_*x*_	*VO*_2_
**2**	Neuron model	mFHN	SRM neuron	LIF
**3**	Number of model parameters	16	8	11
**4**	Internal dynamics of the memristive devices	Yes	No	No
**5**	Experimental validation	Yes	No	No
**6**	Statistical data	Yes	No	No
**7**	Number of memristive devices	2	1	1
**8**	Operating voltage range, V	4 - 7	4	2

As shown in Table 1, the proposed model offers several advantages. It was more flexible possessing 16 independent parameters that offered better opportunity for fine tuning the desired dynamical mode. Next, it permitted an experimental validation using physical memristive devices supported by statistical data. We also employed two memristive devices imitating different channels making the model more attractive in terms of its biological plausibility. Technically, our model demonstrated wider voltage range for the memristive devices what was also important for the model tuning. Finally, we believe that our model could be an appropriate candidate in the development of large-scale non-linear oscillators using memristive devices. Further exploring the model in theoretical part, we will focus on research into chimera states and analysis of potential chaotic oscillation modes. In technical development, particular attention will be given to integrated implementation. This is expected to lead to reduced energy consumption and increased stability, primarily due to the miniaturization of microscale memristors and other circuit components.

## 5 Conclusion

We proposed a mathematical and experimental model that simulates neuronal excitability and synaptic potentiation. The model was implemented by counter-parallel connection of memristive devices with different electrode compositions based on *Au*/*Ta*/*ZrO*_2_(*Y*_2_*O*_3_)/*Pt*/*Ti*/*glass* and *Au*/*Ru*/*ZrO*_2_(*Y*_2_*O*_3_)/*Pt*/*Ti*/*glass* of the mFHN generator circuit. The memristive devices had reliable characteristics possessing stable and gradual bipolar type resistive switching. To describe the model, we proposed a three-dimensional system of non-linear equations that describes three dynamic modes corresponding to excitable, self-oscillatory, and bistable neuronal dynamics. We have discovered that the system can demonstrate burst activity at a certain value of the ε parameter and the inbound external rectangular signal. A hardware implementation of a postsynaptic neuron model based on two counter-parallel memristive devices was developed. We verified computational modeling results with physical prototype memristive neurons in various modes: oscillations and multiple spike activity. We found that in addition, the simulation of neuronal excitability, using two counter-parallel memristive devices, can also model the effect of synaptic potentiation. The effect synaptic potentiation is manifested in the increase of the current amplitude in response to a series of pulse stimulations with constant amplitude. In other words, the memristive circuit proposed in the study can reproduce synaptic plasticity which is the basic feature of connectivity in the brain's neuronal circuit responsible for the generation of higher cognitive functions.

## Data Availability

The datasets presented in this study can be found in online repositories. The names of the repository/repositories and accession number(s) can be found in the article/Supplementary material.
