# Supplementary material for: Memristor-based model of neuronal excitability and synaptic potentiation
Source: Front Neurosci. 2024 Nov 18;18:1456386. doi: 10.3389/fnins.2024.1456386 (PMC11609164; doi:10.3389/fnins.2024.1456386)
Supplement: Supplementary file 1 [file Data_Sheet_1.pdf]

1. Table of parameters of memristive devices used in the manuscripts.

Parameter \ Devices	Au/Ta/ZrO_2(Y)/Pt/Ti/grass	Au/Ru/ZrO_2(Y)/Pt/Ti/grass
γ	0.821	0.582
σ	$0.35 \cdot 10^{-3}$	$0.22 \cdot 10^{-3}$
B	$6.5 \cdot 10^{5}$	$2 \cdot 10^{4}$
b	1.7	0.2
E_b	44.04	39.32
A	10^{8}	
E_m	-36.73	-37.91
δ	29.02	27.05
p	20	
S_{el}	$1.3 \cdot 10^{-7}$	
d	1000	4300
V_{Set}	1	4
V_{Reset}	-1.4	-2

2. The program code used to study the 3D-dimensional system of equations is located at:

https://github.com/Kipelkin/Neuromem

3. Statistical data for memristive devices based on Au/Ta/ZrO_2(Y)/Pt/Ti/grass and Au/Ru/ZrO_2(Y)/Pt/Ti/grass.

a. For Ta:

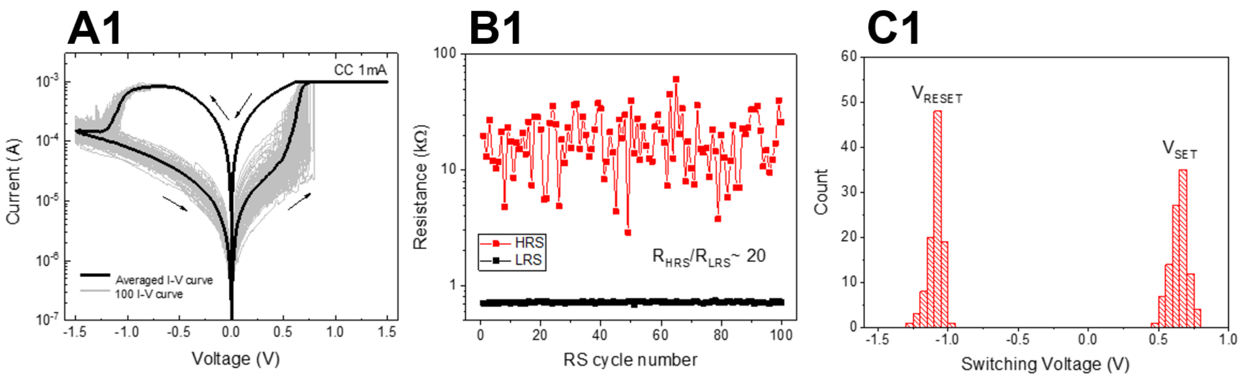

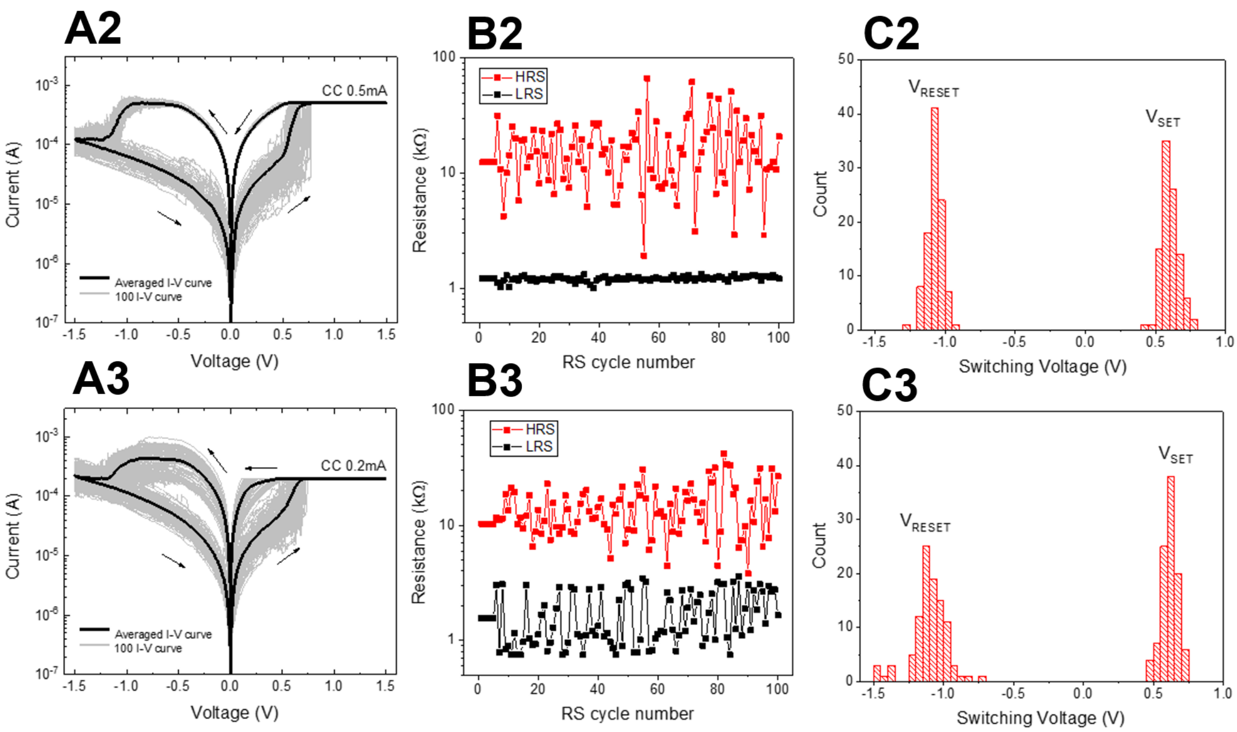

Figure 1. Experimental statistical data for memristive device Au/Ta/ZrO_2(Y)/Pt/Ti/grass. (A1), (A2), (A3) The experimental current-voltage characteristics of non-volatile devices. (B1), (B2), (B3) Resistance values for each switching cycle. (C1), (C2), (C3) Histogram of the resistive states of a memristive device at different switching voltages.

Endurance

Endurance tests were conducted using the National Instruments USB-6341 software-hardware complex. The memristor was sequentially connected to a load resistor (100 Ω) and a current limiter (300 μA) based on a field transistor with a controlled *p-n* junction. A signal consisting of alternating positive and negative switching pulses (with an amplitude of 1.5–2.0 V) and read pulses (0.25 V) was applied to the memristor.

Devices with a *Ta* electrode was tested for endurance under repeated switching cycles. All of them endured over 10^6 switching cycles in pulse mode. The figure shows the dependence of the current states I_{LRS} and I_{HRS} on the number of switching cycles in the Au/Ta/ZrO2(Y)/Pt/grass device, demonstrating 4 million switches with no noticeable degradation of parameters (maintaining a visible window between states) and no failures.

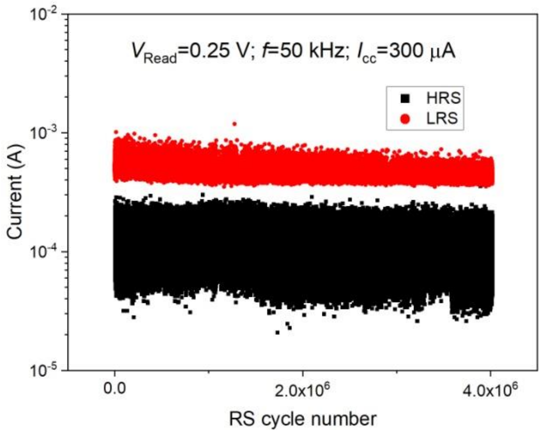

Figure 2. Endurance tests for memristive device

b. For Ru

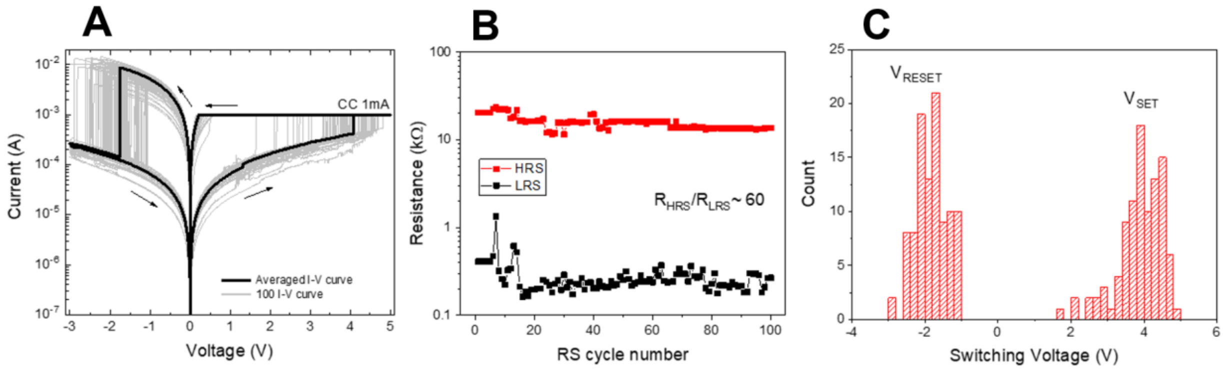

Figure 3. Experimental statistical data for memristive device Au/Ru/ZrO_2(Y)/Pt/Ti/grass. (A) The experimental current-voltage characteristics of non-volatile devices. (B) Resistance values for each switching cycle. (C) Histogram of the resistive states of a memristive device at different switching voltages.

4. Derivation of the equations (1) of the modified memristive neuron FHN.

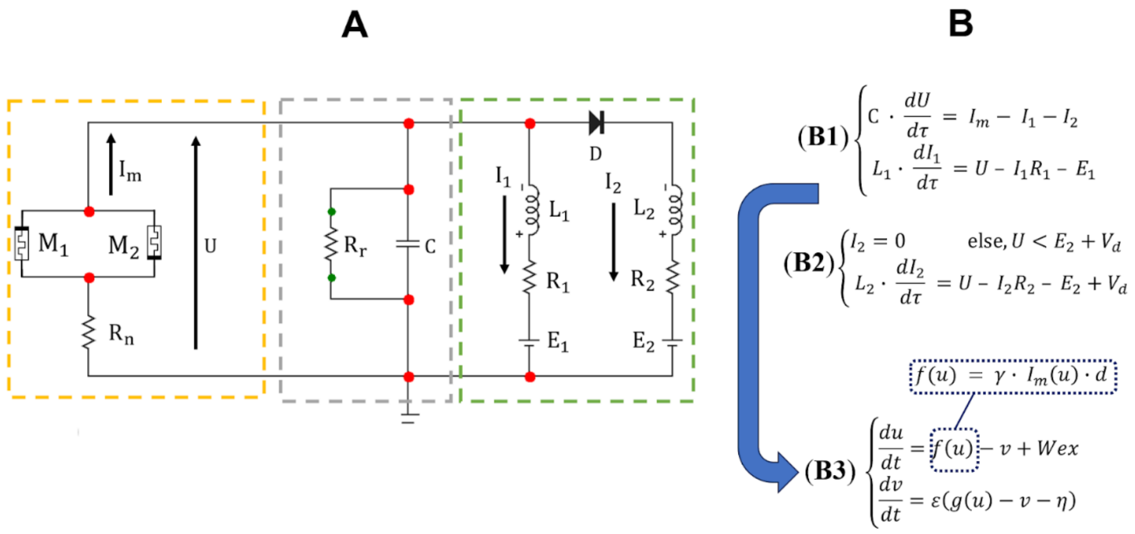

Figure 4. A structural electrical circuit based on memristive devices (A) and equations (B) obtained during the application of Kirchhoff's law (B1) and (B2), as well as their dimensionless form (B3).

5. Figure 5 shows a graph of the dependence of the difference in peak current values (ΔI) of memristive devices on the migration barrier for ions (φ_{ion}), which, by its nature, is contained in the correction parameter b.

$$b = \frac{\varphi_{ion} \cdot q}{kT}, \ \varphi_{ion} = [0.8\,;1]$$

q - charge. k - Boltzmann constant, T - temperature in Kelvin.

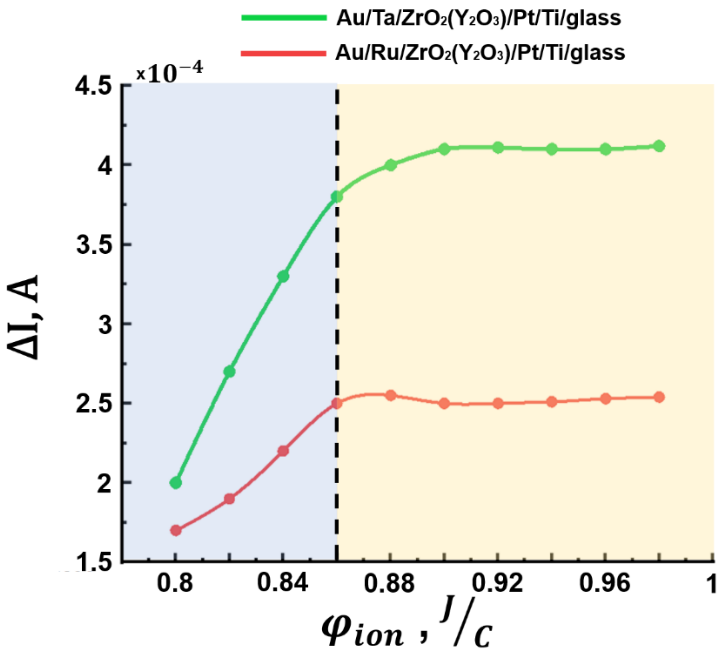

Figure 5. The dependence of the difference in peak current values in memristive devices on the migration barrier for ions.

Based on the figure, a gradual increase in dependence can be observed (proportionally following a linear law) until a certain critical value is reached, after which the dependence remains constant. Furthermore, this critical value differs for each device. Each memristive device has its own critical value for the potential barrier of ion migration, after which the relaxation processes occurring inside reach saturation.

6. Photographs of the experiment and the installation for determining the characteristics of memristive devices.

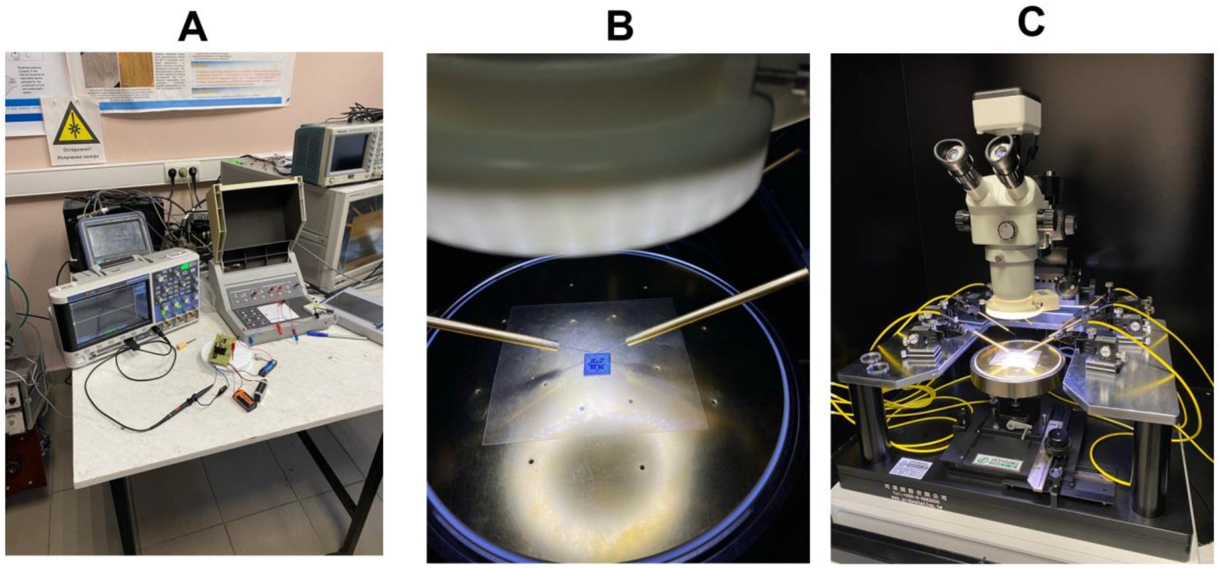

Figure 6. The photographs of experimental installations: (A) The experiment process, (B) Memristive chip, (C) Installation for connecting memristive devices to the FHN generator.
